# Male breast cancer arising in ectopic axillary breast tissue: A diagnostic dilemma

**DOI:** 10.3892/ol.2013.1300

**Published:** 2013-04-11

**Authors:** YANGCHUN XIE, JIN HUANG, DESHENG XIAO, MEIZUO ZHONG

**Affiliations:** 1 Departments of Oncology, Central South University Xiangya Hospital, Changsha, Hunan 410008, P.R. China; 2 Pathology, Central South University Xiangya Hospital, Changsha, Hunan 410008, P.R. China

**Keywords:** male breast cancer, ectopic axillary breast tissue, diagnosis

## Abstract

Male breast cancer arising in ectopic axillary breast tissue is a rare occurrence and few cases have been reported in the literature. Due to its rarity, male axillary breast cancer is easy to misdiagnose. As for adenocarcinoma in the axilla, it is difficult to identify whether the origin is the mammary tissue or the skin appendages, particularly in cases where there is a poor differentiation. The present study reports the case of a male patient with a right axillary lesion that had been present for 6 months. A histological evaluation revealed the features of a poorly-differentiated adenocarcinoma with regards to the pathological report. The patient was diagnosed with a metastatic adenocarcinoma with unknown primary origin. However, following 4 cycles of intensive chemotherapy, the patient experienced bone metastasis while the local lesion was in partial remission. Further immunohistochemistry confirmed its mammary origin. There is limited literature relating to male ectopic axillary breast cancer, and a high probability of misdiagnosis of this disease.

## Introduction

Male breast cancer is of rare occurrence compared to female equivalent. The peak age of incidence for males is 71 years, while for females it is 52 years (1). Although rare, male breast cancer usually has the same common presentation as female breast cancer. However, male breast cancer arising in ectopic axillary breast tissue is an extremely rare malignant neoplasm that has a high incidence of misdiagnosis. Due to the atypical location, a correct diagnosis is often reached during the later stages of cancer. The number of previous studies on male axillary ectopic breast cancer is extremely low and we have found only one relevant case report (2). In the current study, a rare case of male breast cancer arising in ectopic axillary breast tissue is presented, highlighting specific issues with the diagnosis and treatment of this pathology. Written informed consent was obtained from the patient.

## Case report

### Clinical presentation and diagnosis

**Figure 1:**
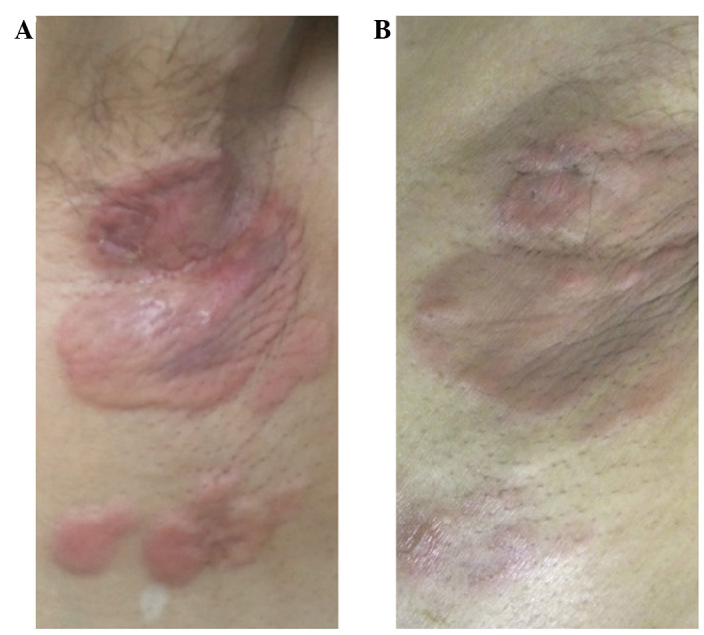
(A) Pre- and (B) post-therapeutic appearance of the right axillary lesion.

**Figure 2:**
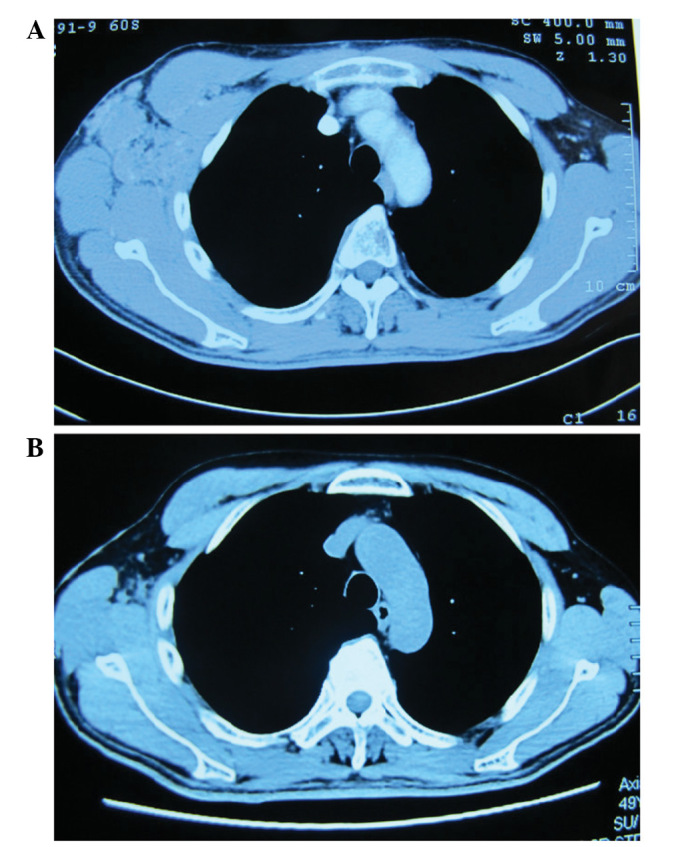
Computed tomography image of the complete right axillary neoplasm with surrounding tissues (A) prior to and (B) following therapy. The images reveal that the tumor reduced in size following 4 cycles of chemotherapy with paclitaxel and cisplatin.

A 51-year-old Chinese male presented with a right axillary superficial mass that had been present for 6 months. The lesion was slow growing and asymptomatic. A physical examination revealed a seemingly healthy male with multiple areas of erythema and several swollen lymph nodes in the right axilla. No mass was palpable inside either breast. The skin lesion was of an irregular shape and size, with maximum dimensions of ∼6×4 cm. With the exception of a reddish appearance, there was no ulceration, swelling, pain or fever of the skin (Fig. 1A). The physical examination also revealed firm, non-tender, enlarged lymph nodes in the right axilla. No cervical, supraclavicular or contralateral axillary swollen lymph nodes were observed. Notably, the patient underwent a right axillary lesion biopsy prior to admission and the pathology report revealed a poorly-differentiated adenocarcinoma. The immunohistochemistry analysis found that the tissue sample was positive for CK-7 and CK-Pan and negative for gross cystic disease fluid protein 15 (GCDFP-15), mammaglobin, the estrogen and progesterone receptors and S-100 protein, indicating that the deformed cells were of epithelial origin. To exclude a metastatic adenocarcinoma, a series of analyses were performed. A C-12 laboratory study demonstrated that the carcinoembryonic antigen (CEA) levels were high at 73.47 ng/ml (normal level, <5 ng/ml). A computed tomography scan of the thorax (Fig. 2) revealed a 5.6×4.1-cm mass in the right axilla, with enlarged adjacent lymph nodes, while the contralateral axilla was normal. The results of a gastrointestinal endoscopy were normal. In addition, the patient underwent positron emission tomography-computed tomography in which the right axilla was observed to exhibit an increase in ^18^F-fludeoxyglucose (^18^F-FDG) uptake. Based on the clinicopathological and ancillary test results, the patient was diagnosed with a metastatic adenocarcinoma of unknown primary origin.

### Treatment and clinical course

**Figure 3:**
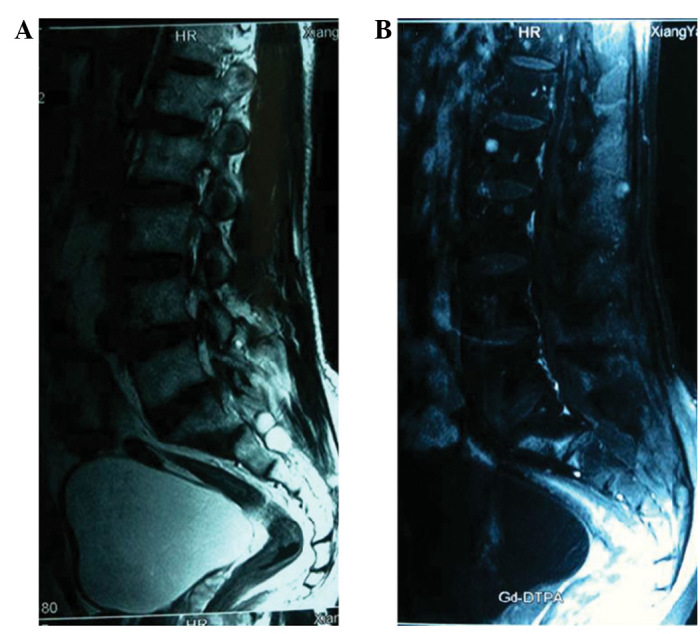
(A) MRI and (B) contrast-enhanced MRI revealing osteogenic bone metastases in the spine.

**Figure 4:**
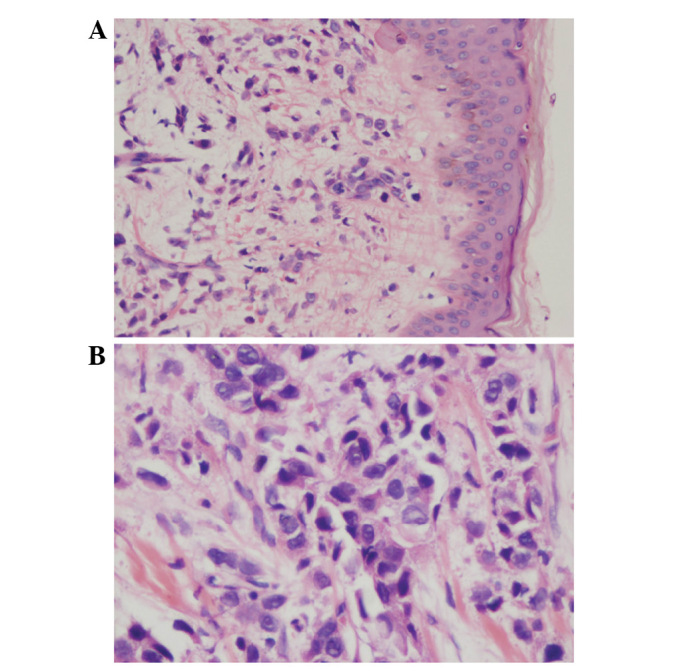
Images of HE staining of histological sections of the lesion from the right axillary at (A) ×200 and (B) ×400 magnification. The majority of the small tumor cells are ovoid- and polygon-shaped, the cytoplasms are stained with basophilic blue and have no or few vacuoles, the cell nuclei are round or ovoid and the chromatin is unevenly distributed.

**Figure 5:**
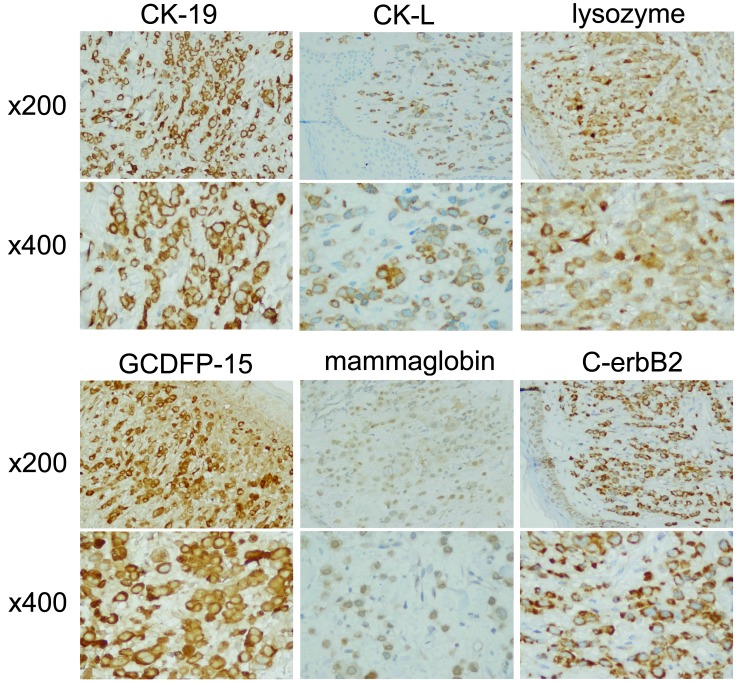
Expression of CK-19, CK-L, lysozyme, GCDFO-15, mammaglobin and C-erbB2 in the excisional specimen of the right axilla. Positive signals (in brown) were detected on the membrane of tumor cells (magnification, ×200 and ×400 as indicated).

The patient received chemotherapy cycles of paclitaxel (175 mg/m^2^) and cisplatin (75 mg/m^2^) every 21 days. After four cycles, the patient was referred to the Central South University Xiangya Hospital (Changsha, Hunan, China) with new lesions (Fig. 1B) and distant metastasis, as shown by MRI (Fig. 3). Progression within a short time may have been indicative of an initial misdiagnosis or tumor resistance to the therapy. As a consequence, an additional biopsy was performed. The pathological report once again diagnosed a poorly-differentiated adenocarcinoma involving subcutaneous tissue (Fig. 4). Macroscopically, no tumor nests were found and no crypt-like structures were identified in the background. The specimen consisted of skin and subcutaneous tissue. An immunohistochemistry analysis revealed that the dissected tissue was positive for CK-19, CK-L, lysozyme, GCDFP-15, mammaglobin and C-erbB2 (Fig. 5). The overexpression of C-erbB2 markedly indicated that a breast adenocarcinoma could not yet be ruled out. The immunohistochemical analysis of melan-A, HMB45, TG, CT, PSA, PSAP, P504S, TTF-1, napsin, myogenin, actin, HHF35, CDX-2, P63 and vimentin was also performed (results not shown) and the specimen was observed to be negative for these markers. This eliminated melanoma, thyroid gland, prostate, lung, muscle, intestine and squamous epithelium origins, respectively. A diagnosis of carcinoma arising in the ectopic breast tissue of the axilla was then made. A follow-up of this case was not performed due to personal reasons given by the patient, however, the case has been discussed here as it is important for aiding in the improvement of clinical performance, including forming a diagnosis and the pathological analysis.

## Discussion

Male breast cancer is extremely rare compared with female breast cancer. Ectopic breast tissue has been identified in a number of regions, including in the vulva (3), anal polyps (4), axilla (5,6) and axillary lymph nodes (7), affecting up to 6% of the general population and occurring more frequently in females and the axillary region (8). Thus, the occurrence of male axillary breast cancer is extremely uncommon and to the best of our knowledge, only one case of male axillary breast cancer has been reported (2). The presentation of male axillary breast cancer may have a wide differential diagnosis and, in particular, metastatic carcinoma from the breast or other origins must be considered, as well as the diagnosis of a primary origin (9). For the current patient presenting with adenocarcinoma arising from axilla, the first step of the differential diagnosis was to determine whether the mass was a primary lesion of a metastatic epithelial neoplasm from the breast, the gastrointestinal tract, the lung or the prostate. Primary carcinoma arising in the axilla may be difficult to differentiate from metastatic carcinoma of any type of origin and from carcinoma arising in heterotopic axillary breast tissue, as ectopic breast tissue is located in the subcutaneous tissue and deep dermis of the skin, where it often integrates with normal skin appendages (10). Since the axillary region has abundant sweat and sebaceous glands, diagnoses of cutaneous adnexal malignancies must be differentiated between. In this instance, a differential diagnosis is difficult as these two entities are often morphologically indistinguishable. In the present case this was particularly apparent as the adenocarcinoma was poorly-differentiated, making morphological distinction extremely difficult. Therefore, in the present case, immunohistochemical analysis was performed to generate a reliable diagnosis. A previous study reported that accessory breast carcinoma is diagnosed in the same manner as anatomical breast carcinoma using a physical examination and ancillary tests, followed by a pathological diagnosis via fine needle aspiration cytology, gross excision or any other type of biopsy (11). To the best of our knowledge, a panel of immunohistochemical markers, including the estrogen and progesterone receptors and C-erbB2, are more useful in the pathological diagnosis of mammary carcinoma. In addition, GCDPF-15 and mammaglobin represent significant immunomarkers of breast cancer. Lewis *et al*(12) found that mammaglobin is a more sensitive, but less specific, marker of breast cancer compared with GCDPF-15, as mammaglobin is not only expressed in breast carcinoma tissue but also in benign breast epithelium, while normal breast tissue does not express GCDPF-15. In addition, mammaglobin-A has previously been used for the non-invasive, *in vivo* detection of cancerous cells in mouse breast cancer with metastatic axillary lymph nodes and has shown a high resolution and specificity, indicating a potential for future translation into the provision of clinical guidance (13). Serra *et al*(14) reported that lysozyme-positive male breast cancer has an unfavorable outcome.

In the present case report, immunohistochemistry was used to generate a diagnosis of male breast cancer arising in the ectopic axillary breast tissues and to fully exclude other types of origin and common presentations of breast cancer metastasis to the axilla.
